# Are politically diverse Thanksgiving dinners shorter than politically uniform ones?

**DOI:** 10.1371/journal.pone.0239988

**Published:** 2020-10-27

**Authors:** Jeremy A. Frimer, Linda J. Skitka

**Affiliations:** 1 Department of Psychology, University of Winnipeg, Winnipeg, MB, Canada; 2 Department of Psychology, University of Illinois at Chicago, Chicago, IL, United States of America; Middlesex University, UNITED KINGDOM

## Abstract

Americans on the political left and right are engaged in a Culture War with one another, one that is often characterized by mutual fear, antipathy, and avoidance. Are there safe havens from the socially straining effects of this Culture War, times and places where Americans of different political stripes gather and put aside their political differences? Previous research (Chen & Rohla, 2018) implied that there might not be insofar as even intimate family gatherings seem to be subject to Culture War tensions. They found that politically diverse Thanksgiving Dinners were 35–70 minutes shorter than politically uniform ones, representing a 14–27% reduction in overall dinner duration. Noting analytical and methodological limitations in the prior analysis, we conducted two pre-registered studies to test whether diverse dinners are shorter than uniform ones and to attempt to conceptually replicate and extend this prior analysis. Individual analyses yielded mixed results, with null models generally supported but effect estimates generally overlapping with those of Chen and Rohla (2018). A mega-analysis found that, when controlling for various covariates, politically diverse dinners were 24 minutes shorter than politically uniform ones, 95% confidence interval = [9, 39], representing a 6% decrease in the total dinner time [2%-10%]. This final result successfully replicates Chen and Rohla (2018) both in terms of effect overlap and direct-and-significance criteria while nonetheless favoring the conclusion that politics is not straining family ties as much as previously thought.

## Introduction

The United States is in the midst of a Culture War [1, 2]. The majority of politically engaged citizens report that they feel afraid of and anger toward members of the other party. This interpartisan animosity appears to be a growing phenomenon; the percentage of partisans who reported “very unfavorable” views of the other side more than doubled in the past two decades, from ~20% in 1994 to ~55% in 2016 [3]. The rift has become so strong that it seems to now be stronger than racial, religious, or ethnic tensions [4]. Both sides are motivated to avoid the other side, even showing a willingness to forgo money to avoid hearing what the other side has to say [5]. These sentiments are strong enough that they can manifest as discrimination at work [6] and in relationships [7]. The result is spatial sorting, with people tuning in to ideologically congenial news outlets, echoing the sentiments of likeminded others in social media bubbles [8, 9], and moving to politically homogenous neighborhoods [10].

The psychological motives that lead to spatial sorting are at least twofold [5]. First, hearing from unlike-minded others means that a person consumes information that conflicts with their existing beliefs. This causes the observer to experience cognitive dissonance, which tends to be accompanied by an aversive feeling [11–13]. Second, agreeable conversations with others about important beliefs and values satisfies a fundamental human need to belong [14] and for a shared sense of reality [15], whereas tense disagreements threaten the same. These theorized and previously empirically supported mechanisms set out the specific conditions that are most likely to induce spatial sorting. Simply being in close proximity to unlike-minded others might not necessarily cause sorting; however, when people talk about ideological and political topics, the social atmosphere should become unpleasant and this sentiment should drive unlike-minded people away from one another.

The question that motivates this research concerns whether there are limits to this political rift in the form of times and places where people set aside their political differences and get along for the sake of enjoying an event together. A prime candidate for such a truce might be Thanksgiving dinner, a traditional family gathering in which relatives come together to enjoy a meal and one another’s company, and to feel gratitude for what they have. Norms concerning polite company proscribe conversations about politics (as well as sex and religion), making Thanksgiving a prime candidate for suppressing relationship-straining political conversations in politically diverse company for the sake of getting along.

The available evidence, however, suggests that Thanksgiving dinner is not immune to the Culture War between liberals and conservatives. Chen and Rohla [16] analyzed ~25 billion smartphone location data pings of ~10 million Americans in 2015 and 2016 to reach the conclusion that politically diverse Thanksgiving dinners were “30 to 50 minutes shorter” (p. 1020) than politically uniform ones, a substantial 12–19% reduction of the total dinner time (*M =* 257 minutes).

Chen and Rohla’s [16] innovative recruitment of smartphone location data was ground-breaking insofar as it permitted precise measurement of times and locations and generated a massive data set. These features allowed the researchers to detect potentially small but important effects with precision. Without self-reports or behavioral measures attesting to the political leanings of the participants, Chen and Rohla devised a new and innovative approach to infer the political beliefs of dinner participants. This approach drew inferences from the political leaning of the traveler’s home precinct and of the Thanksgiving dinner location. However, we note (and detail below) that the validity of this inference is questionable, leaving open the broader question about whether political diversity really does shorten Thanksgiving dinners and by how much.

To understand the questionable features of the political diversity measure requires an understanding of the specific methods that Chen and Rohla used. Political diversity is the phenomenon whereby two or more people hold different, mutually oppositional, political beliefs, and is thus tantamount to a mismatch between the political positions of members of the social gathering. For instance, in a dinner event attended by a Democrat and a Republican, diversity is high whereas a dinner with two Democrats has low diversity. An intuitive approach to operationalizing diversity would involve directly assessing the political attitudes of each person in attendance, by asking them or acquiring their voting record for example, and then computing diversity as the degree of mismatch.

Chen and Rohla relied on a different and novel approach to measure political diversity. They inferred the political beliefs of each person from the voting precinct in which their home was located (determined by their location in the middle of the night several days before Thanksgiving) and publicly available information about the general voting tendency in that same precinct in the 2016 U.S. Presidential Election. To understand how this method works and its’ critical limitations that motivate the present work, we present an illustrative, hypothetical example in which a hypothetical person named Joe travels ~25 miles west from his home in downtown Austin, TX (277^th^ precinct) to a Thanksgiving dinner in the western suburb of Austin near Lake Travis (232^nd^ precinct). Knowing nothing more than Joe’s home location and the location of the dinner, the method would infer that the political diversity (probability of a mismatch) of the dinner was .53 on the 0–1 scale.

The method begins by inferring that Joe is probably a Democrat by virtue of the fact that his precinct voted in favor of Hillary Clinton over Donald Trump in the 2016 U.S. Presidential election by a margin of 72% to 21% [17]. That is to say that ideological position was operationalized as a *probability* of being a Democrat (in this case the probability is 77% based on Chen and Rohla’s formula). Meanwhile, the inferred political leaning of a home near Lake Travis in the western suburbs, is computed in the same way and ends up being a 45% probability of being Democratic (The 232^nd^ precinct favored Trump over Clinton by a margin of 52% to 42%; [17]). The mismatch was then computed as a function of these probabilities of 77% and 45% to arrive at a relatively high diversity estimate of .53 (see Chen and Rohla [16], for the formula), which was approximately 1 *SD* above the *M* diversity in Chen and Rohla’s sample.

In contrast, Joe’s next-door neighbor Alice traveled to a dinner approximately the same distance from downtown but toward the south near Bluff Springs (413^rd^ precinct). Alice’s dinner would have a relatively low diversity score of .33 (~ 1 *SD* below the *M* in Chen and Rohla) because like 277^th^ precinct downtown, the 413^th^ delivered 75% of the vote to Clinton and much less (18%) to Trump.

We note several limitations with this method. An analytical limitation was that Chen and Rohla [16] extrapolated beyond the populated range of the data to conclude that diversity shortens Thanksgiving dinner by 30–50 minutes, which would mean that the 30–50 minute figure might be an over-estimate. This was accomplished by plotting political diversity (x) against dinner duration (y) and taking the unstandardized slope from a regression analysis as the estimate of the dinner-shortening effect of political diversity. Because the slope reflects the change in dinner duration *for every 1-unit change in political diversity*, this analytical decision effectively compares the duration of dinners with no diversity (0.00) and dinners with full diversity (1.00). However, very few dinners met these descriptions. The average diversity score across all dinners in Chen and Rohla [16] was .44 with a *SD* = .10, meaning that ~68% of all dinners had a diversity score between .34 and .54. Chen and Rohla thus drew inferences about the dinner-shortening effect of diversity by effectively comparing dinners that were 4.4 *SD*s below the *M* with dinners that were 5.6 *SD*s above the *M*, which amounts to an extrapolation beyond the populated range. The 30–50 minute estimate thus described the difference in dinner duration of two hypothetical dinners that virtually never occurred.

In personal communication (June 8, 2016), Chen and Rohla suggested that although political diversity was measured using a continuous measure (ranging from 0–1), the event that the measure aimed to model was a dichotomous event: either the two persons at dinner voted for the same presidential candidate in 2016, or they did not. This *latent* dichotomy would have justified the extrapolation to the theoretical points of interest, namely diversities of 0 and 1.

Frimer and Skitka [18] challenged the claim that Thanksgiving dinner diversity is a dichotomous construct, maintaining that political diversity is more accurately thought of along a continuum. The dichotomous claim requires Thanksgiving dinners be a dyadic event, with just two participants, and that both of them are Democrats and/or Republicans. We suggest that neither of the empirical premises are sustainable. The vast majority of Thanksgiving dinners (98% by our estimates) have more than two people present, with an average of 7 people in attendance (see our Studies 1 & 2). Thus, each dinner has some combination of Democrats and Republicans (e.g., 2 Democrats and 5 Republicans), making the amount of political diversity therein fall on a continuum. Moreover, many Americans did not vote for either Trump or Clinton in the 2016 election. Some 58% of the U.S. population and 44% of eligible voters did not vote in 2016 [19], and 6% of voters chose a candidate other than Trump or Clinton. These sizable non- and third-party voter populations represent ambiguous additional categories with respect to political diversity, which further undermine the dichotomous claim that would have justified an extrapolation beyond the populated range of the data.

We maintain that combinations of multiple Democrats, Republicans, Independents, and non-voters are more accurately described along a continuum of diversity, rather than in terms of a dichotomy. Accepting that diversity is a continuous construct, and with only Chen and Rohla’s data being available at the time, Frimer and Skitka [18] applied a more conventional procedure for estimating the dinner-shortening effect of political diversity by comparing the duration at *M*_*diverity*_ + *SD*_*diverity*_ to the duration at *M*_*diverity*_−*SD*_*diverity*_; doing so produced an estimate of a 4–11 minute reduction from the average dinner time of 257 minutes, a relatively small 2–4% reduction in total dinner duration.

Chen and Rohla’s [16] modeling of Thanksgiving dinner as a dichotomous event raises further questions about the validity of the measurement of political diversity. Their data attempted to represent the political leanings of two members of a larger dinner event. For the measure to survive this validity threat, the political diversity observed between the traveler and the dinner homeowner would need to be representative of the diversity of all attendees. This has yet to established. Moreover, measuring the political diversity of a dinner from the home location of the traveler and the homeowner assumes that each traveler’s political leanings are representative of his/her home precinct and that each homeowner’s political leanings are representative of his/hers home precinct as well. This may or may not be true. The additional available information about each person’s Thanksgiving dinner might justify modifications to those assumptions but are not accounted for within the Chen and Rohla formula. The limitations of the existing measure and analyses cast doubt over firm conclusions about whether politically diverse Thanksgiving dinners are shorter than politically uniform ones, and if so by how much.

## The present studies

Our primary goal was to test whether politically diverse Thanksgiving dinners were shorter than politically uniform ones in the years 2018 and 2019. Our secondary goal was to conceptually replicate Chen and Rohla’s [16] findings, using different and complementary methods. Whereas Chen and Rohla used location data from smart phones to infer the diversity of dinners and their duration, we used a crowdsourcing methodology, asking participants to self-report the time they arrived and departed from Thanksgiving dinner and to tell us about the people in attendance and their political attitudes; we used these data to infer political diversity. The method we employ avoids some of the limitations of Chen and Rohla’s study (detailed previously). Two pre-registered studies, conducted in 2018 and 2019 respectively, measured political diversity and Thanksgiving dinner duration, tested whether the two are associated, and used these data to estimate the dinner-shortening effect associated with political diversity.

Do our correlational studies meaningfully replicate Chen and Rohla’s analyses? Chen and Rohla interpreted their fixed-effects regression analyses on their longitudinal, observational data as having established a causal effect of diversity on dinner duration. Our approach was different in that we employed an OLS regression analysis while controlling for various confounds that we measured. Our analyses might not appear to be suitable for testing whether Chen and Rohla’s causal effects replicate.

Even noting the methodological differences, we suggest that our correlational observations do offer a meaningful opportunity for a conceptual replication and for two reasons. First, Chen and Rohla’s causal inferences presuppose correlations. Causal effects require that (a) the causal variable is correlated with the effected variable, (b) the causal variable precedes the effected variable, and (c) third variables do not explain the association. If any of the three are unsupported, the causal claim fails [20]. Therefore, our correlational tests have the potential to (dis)confirm the correlational feature of a causal relationship (feature a). Second, we question whether Chen and Rohla’s data justified causal inferences in the first place. Fixed-effects regression analyses allow for causal inference if and only if potential confounds are time invariant between the earlier and later observations (November 2015 to November 2016 in their case). Many important changes took place between Thanksgivings 2015 and 2016, with the most notable being the ascendancy of Donald Trump to the U.S. Presidency. For the time invariance tenet to hold, the psychology of the American electorate would need to have been largely unchanged by the campaign and electoral victory of President Trump. This seems to be a strong and tenuous assumption, raising questions about Chen and Rohla’s causal interpretation. If the time-invariance assumption is relaxed, then the two sets of analyses become conceptually comparable as tests of whether diverse dinners are shorter than uniform ones.

Another potential difference between Chen and Rohla’s study and ours might be the time in the election cycle. Chen and Rohla’s main findings were from 2016, a U.S. Presidential election year whereas our tests did not take place on a Presidential election year. However, one of our studies was in 2018, which was a midterm election year. Voter turnout in both 2016 and 2018 was comparable and around 50%-55%, meaning that political engagement was roughly equivalent. Moreover, Chen and Rohla reported that diverse dinners were shorter than uniform ones in both 2015 and 2016 (see their S1 Table in S1 File, models 1 & 2). Thus, we suggest that the years 2018 and 2019 are suitable to testing the effects that Chen and Rohla observed.

Our criteria for replication success are twofold. The first is direction and significance: do we observe effects that are in the same direction (and reach significance) as those reported in Chen and Rohla? The second replication criterion is (unstandardized) effect overlap. Chen and Rohla estimated the effect of diversity on dinner duration in one zero-order model and three corrected models (using fixed-effects regression to control for covariates), with the former predicting a 22 minute decrease, 95%CI = [19, 24] and the corrected models predicting 38 [35, 41], 45 [37, 53], and 56 [42,70] minute decreases. We use our zero-order correlations to test whether Chen and Rohla’s zero-order effect replicates and corrected correlations (controlling for covariates) to test whether the latter three replicate. To conservatively test for replication of the corrected estimates, we take the minimum and maximum estimates from the three analyses in aggregate [35, 70] as the target 95% confidence interval. We also test whether our effects replicate Frimer and Skitka’s [18] interpretation of Chen and Rohla’s [16] data, which yield zero-order estimate of a 4–5 minute reduction and a corrected estimate of a 7–14 minute reduction.

A secondary goal was to extend Chen and Rohla [16] by proposing and testing a social psychological explanation for the shorter dinners. Chen and Rohla’s [16] explanation for the effect was sociological, implicating the role of political advertisements. Our interpersonal level of analysis led us to theorize that political diversity shortens Thanksgiving dinner because talking about politics induces tension and conflict between un-likeminded people. If so, we should expect that diversity will predict shorter dinners especially when the conversation gravitates to politics (a moderator). The corollary would be that politically diverse gatherings might be able to avoid the socially deleterious effects of diversity by avoiding the topic of politics (as politeness rules suggest). We also examined the theoretically implicated affective mediator, that diversity makes the social atmosphere unpleasant and this negative atmosphere in turn shortened dinners.

## Study 1: Thanksgiving 2018

Pre-registered (https://osf.io/3se4z/registrations) Study 1 tested whether politically diverse Thanksgiving dinners were shorter than politically uniform ones in the year 2018. We also examined whether the dinner-shortening effect of political diversity was especially strong when the conversation gravitated to politics, and whether diversity soured the social atmosphere, which in turn reduced the dinner duration. We recruited a sizable sample in the days leading up to Thanksgiving dinner 2018 and provided instructions to record details about the dinner (Part I); we then invited these people to report their observations after the dinner in Part II.

We operationalized political diversity in relation to attitudes toward President Trump. Our reasons for doing so were to, as closely as feasible and reasonable, follow the methods of Chen and Rohla [16]. They operationalized diversity in relation to whether people voted for Donald Trump or Hillary Clinton in the 2016 U.S. Presidential election. With the election two years in the past in 2018, Hillary Clinton was a less prominent public figure; public attention was more focused on the President Trump. Moreover, whether or not Americans approved of President Trump was a good first approximation of which side of the Culture War they endorsed: at the time of the study, Trump’s approval among people who voted for Hillary Clinton in 2016 was just 10% [21], 12% among Democrats, and 85% among Republicans.

### Method

#### Participants

Conducting a power analysis from Chen and Rohla [16] was complicated by the wide range of effect sizes reported (model *r*s = -.02, -.26, -.68, and -.81) depending on the various models used. An alternative approach, and the one we adopt here, is to leverage the proposed explanation: Thanksgiving dinners are thought to be shorter when there is political diversity because of selective exposure motivation, the general desire to avoid hearing from the “other side.” Frimer, Skitka, and Motyl [5] estimated an effect size of *r* = -.30 in their study of people’s preference to speak with like-minded (compared to unlike-minded) people on social and political issues. Given that the duration of Thanksgiving Dinner does not hinge solely on selective exposure motivation (other factors are likely at play as well), we conservatively assume an effect size half the size observed by Frimer et al., or *r* = -.15. We would therefore need 580 Americans for 95% power to detect the predicted correlation between diversity and dinner duration (*r*_estimated_ = -.15). Our pre-registered goal was therefore to recruit 1500 participants in the preliminary Part I with hopes of retaining a final sample of at least 580 (Part II).

#### Procedure

This study was reviewed and approved by the University Human Research Ethics Board at the University of Winnipeg (HE12152). Part I was posted on Mechanical Turk on November 20, 2018—two days before Thanksgiving 2018 (November 22). In Part I, payment was $0.10. Participants reported if and when they planned on having Thanksgiving Dinner, received instructions to take note of the *precise* times that they arrive and leave, and then to complete Part II the day after Thanksgiving. We invited those who completed Part I to complete Part II using TurkPrime. As was our pre-registered plan, we stopped recruiting Part II participants once we had 580 participants. This left us with 581 respondents. We excluded 2 participants who in Part II reported that they did not have Thanksgiving dinner after all, leaving *N* = 579.

In Part II, payment was $1.00. Participants reported how many people were at Thanksgiving Dinner, then reported each of those individuals’ attitude toward President Trump. Next, they indicated whether and where they had Thanksgiving Dinner, the time that they arrived and left, as well as provided a confidence estimate surrounding their arrival and departure times. They then reported the topic of conversation at dinner and how pleasant the social atmosphere was. Finally, they supplied demographic information and received a debriefing.

*Part I*. A question asked, “Do you plan on having Thanksgiving Dinner with other people this year?” Response options were *yes* and *no*. Most (92%) responded in the affirmative. Only those participants that selected yes were then asked, “When?” with response options being *Thurs Nov 22*, *Fri Nov 23*, *Sat Nov 24*, *Sun Nov 25*, and *other*. The most common date was the Thursday (85%), with 2% each having dinner on the Friday, Saturday, and Sunday and 1% indicating some other date. They then read the following:

You have qualified for Part II! In this study, we are interested in the details of your experience at Thanksgiving Dinner. On the day of Thanksgiving Dinner, please take note of precisely when you arrive and when you leave the house where Thanksgiving Dinner takes place. If possible, record the time down to the minute. Please make a note to yourself now (for instance, in your calendar) to remind yourself to record the time you arrive and leave from Thanksgiving Dinner on the day of. The day after your dinner, a “Thanksgiving Dinner Part II” study will appear on Mechanical Turk. It will pay $1.00 for about 5 minutes of your time. Having completed Part I, you will have privileged access to this study. Please look for and take “Thanksgiving Dinner Part II” once it becomes available.

*Part II*. In Part II, participants were asked the following questions.

*Dinner location*. The question asked, “where did you have Thanksgiving dinner?” Response options were: *I didn’t have Thanksgiving dinner* (0.3%), *at my place* (42.8%), *at a restaurant* (4.0%), and *at someone else’s place* (52.7%). The final option allowed participants to specify where in a text box (not analyzed). We excluded the two participants who indicated that they did not have Thanksgiving dinner after in all subsequent analyses.

*Duration*. We asked participants to report the time they arrived and departed from Thanksgiving Dinner. The arrival question asked, “What time did you arrive at Thanksgiving dinner? If you had dinner at home, what time did the first guest arrive? Please use the format hh:mm am/pm (e.g., 4:35pm), and be as precise as possible—down to the minute if you know.” Some participants did not follow the specified format, but we were able to ascertain the time in all instances. S1 Fig in S1 File displays histograms of the arrival and departure times. We computed the duration of Thanksgiving dinner by subtracting the arrival time from the departure time (*M* = 313 minutes, *SD* = 162 minutes, range: 30–1225 minutes). Thanksgiving gathering durations were normally distributed (skew = 1.40; see S2 Fig in S1 File).

After reporting a time, participants reported their confidence in their reported time by completing the statement, “I feel confident that this time is…” Response options were *within 1 minute of when I actually arrived* (20.6%), *within 5 minutes of when I actually arrived* (41.6%), *within 10 minutes…* (24.5%), *within 20 minutes…* (7.9%), *within 30 minutes…* (3.3%), *within 1 hour…* (1.0%), and *within 4 hours…* (0.9%). They were then asked analogous questions about the time they left Thanksgiving dinner. We counted the least accurate confidence estimate to be each person’s confidence estimate (reported above in parentheses).

*Topic of conversation*. Participants reported the topic of conversation. A question asked, “How often did the conversation center on…?” For each of *politics*, *sports*, *work*, *friends & family*, and *food*, participants responded on a 101-point scale anchored at 0 (*rarely*), 33 (*once in a while*), 67 (*often*), and 100 (*constantly*). In descending order, the most common topics of conversation were *family and friends* (*M* = 59, *SD* = 25), *food* (*M* = 58, *SD* = 26), *work* (*M* = 29, *SD* = 22), *sports* (*M* = 28, *SD* = 27), and *politics* (*M* = 14, *SD* = 18). Following our pre-registered plan, we computed a talking-about-politics score by subtracting the average of *sports*, *work*, *friends & family*, and *food* from *politics* and computing z-scores.

*Pleasant social atmosphere*. Participants described how pleasant the social atmosphere was by responding to the question, “How was the social atmosphere at dinner?” There were 6 items (*pleasant*, *relaxed*, *happy*, *tense*, *sad*, and *offensive*) and the 101-point scale was anchored at 0 (*not at all*), 33 (*slightly*), 67 (*somewhat*), and 100 (*very*). Overall, the social atmosphere was favorable with high scores on the three positive elements (*M*s between 77 and 79, *SD*s between 22 and 23) and low scores on the three negative elements (*M*s between 5 and 8, *SD*s between 11 and 15). Reverse scoring the negative elements, we computed a pleasant social atmosphere aggregate score, *M* = 86, *SD* = 13, α = .80.

*Political diversity*. We measured political diversity in four steps. First, participants reported how many people were at dinner. The question asked, “How many people were at Thanksgiving dinner 2018 (not including yourself)?” Participants answered on a 10-point scale anchored at *1*, *2*, *3*, …, *8*, *9*, *10+*. For simplicity, we scored *10+* as 10. We added 1 (the self) to the reported number to constitute the measure of attendees. The average dinner had 7.18 attendees (*SD* = 2.83). Just 1.9% of Thanksgiving dinners had 2 attendees. Second, participants identified the attendees by name. The question asked, “What are the names of the people who were there? Please provide the first names and last initials (e.g., Mike G).” There were spaces to report names for each of the attendees, up to 10. Third, participants reported the political attitudes toward President Trump of each of the attendees. The question asked, “Does {name} approve or disapprove of President Trump?” with an attendee’s name replacing {name} in each instance. Response options were *disapprove*, *neutral*, *approve*, and *I don’t know*. A final question asked, “Do you approve or disapprove of President Trump?” and presented the same response options. Participants were not forced to report the attitudes of each person and 47% of participants did so for all attendees. On average, participants reported the attitudes of 6.51 attendees, including themselves (*SD* = 2.75). Fourth, we computed a political diversity (or “mismatch”) at Thanksgiving Dinner score (possible range 0 to 1) using the pre-registered Eq 1.

Diversity=D×A+0.5(D+A)(N+U)n!2(n−2)! (1)

Where…

*D* = # people who disapprove of Trump

*A* = # people who approve

*N* = # people who are neutral about Trump

*U* = # people whose attitude toward Trump is unknown

*n* = # people described at dinner (maximum possible = 10+self = 11)

This equation computes the average mismatch of all the dyads at dinner and makes the following assumptions: (a) people who approve and disapprove are “fully” mismatched (mismatch score = 1); (b) people who approve and people who are neutral are partially mismatched (mismatch score = 0.5). The same applies for *D*s and *U*s, *A*s and *N*s, and *A*s and *U*s; and (c) *D*s and *D*s are not mismatched (mismatch score = 0). The same applies for *A*s and *A*s, *U*s and *U*s, and *N*s and *N*s. Table 1 describes several real examples of the calculation of diversity scores.

**Table 1:** Real examples of the compositions of Thanksgiving dinners, 2018, and their associated political diversity scores.

*n* Attendees with Attitudes toward Trump				
Disapprove	Neutral	Unknown	Approve	Diversity
11	0	0	0	.00
0	0	0	4	.00
0	4	1	0	.00
0	0	1	9	.10
0	1	0	4	.20
7	1	0	1	.30
1	0	0	4	.40
6	1	0	3	.50
2	0	0	3	.60
2	0	0	2	.67
1	0	0	1	1.00

The average diversity score was .25 (*SD* = .22), which upon first glance might appear relatively low (ideologically uniform). However, we suggest that mean level reflects a relatively moderate level of diversity. This is because larger dinners have a lower upper limit of diversity scores than smaller dinners. For instance, a dinner of two people can have diversity scores as high as 1.00. But a dinner of 7 people (the average in this study) can have a maximum diversity of .57 (e.g., 3 Trump supporters + 4 Trump opponents). The lower levels of diversity in larger dinners is not an artifact of our operationalization. It also makes sense phenomenologically. In a dinner of 3 supporters and 4 opponents, the supporters will share attitudes with the other 2 supporters present and the same goes among opponents. Everyone will have allies present. In contrast, a dinner of just one supporter and one opponent has only one dyad, which is a mismatch. Chen and Rohla [16] restricted their analyses to dyads, making .00 to 1.00 the working range in their analysis. Our analyses allowed up to 11 dinner participants, making .00 to ~.60 the working range of diversity scores in our analyses. Therefore, average scores of .25 is approximately midway along the working range of possible diversity scores. Both highly uniform and highly diverse dinners were represented in our sample.

Finally, we also computed an overall political leaning of the dinner, with higher scores being more pro-Trump. It was computed as (*A*-*D*)/*n*. This metric allows scores to vary from -1 (everyone against Trump) to 1 (everyone is for Trump) and had a *M* = -.35 (*SD* = .63).

### Results

**Fig 1:**
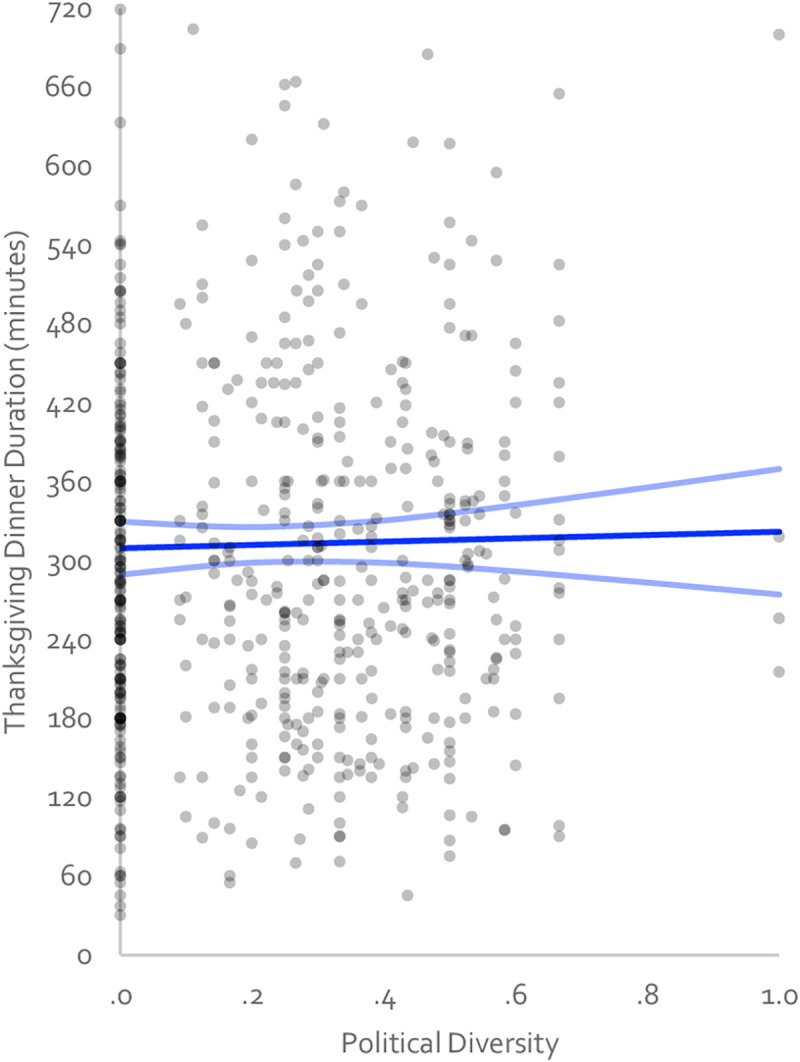
Scatterplot and OLS regression line (with 95% confidence interval) showing the relationship between ideological diversity and Thanksgiving dinner duration in 2018. Each dot represents a Thanksgiving Dinner event.

**Fig 2:**
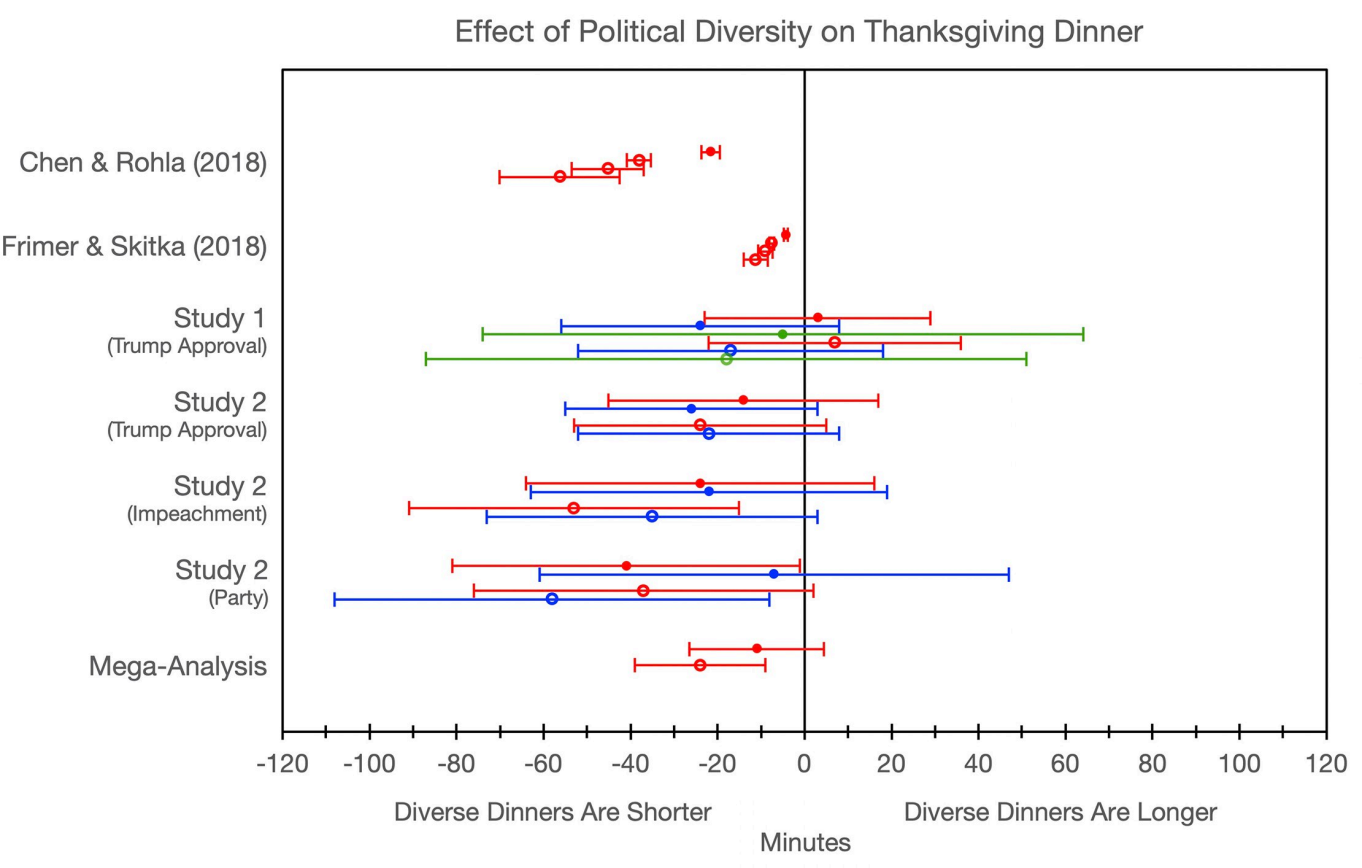
Estimates of the effect of political diversity on Thanksgiving dinner duration in minutes. Red represent analyses with full samples whereas blue and green represent analyses with participants that were confident about their arrival and departure time within 5 and 1 minutes respectively. Filled dots represent zero-order effects whereas open dots represent conditioned correlations while controlling for various covariates. Operationalizations of diversity are in parentheses. Error bars represent 95% confidence intervals.

We first tested whether diverse Thanksgiving dinners were shorter than politically uniform ones with all participants included in the analysis. Our OLS regression with diversity predicting dinner duration did not reach significance (see Fig 1 and Table 2). Our preregistered interpretative strategy was to estimate the effect of diversity on dinner duration by comparing the duration of dinners that were relatively diverse (*M*_diversity_ + 1*SD*_diversity_ = .46) and dinners that were relatively uniform (*M*_diversity_ - 1*SD*_diversity_ = .03). This analysis produced an estimate that diverse dinners were 3 minutes longer than uniform ones, a minor 1% change in the total dinner duration, 95%CI = [23 mins shorter, 30 mins longer, representing a -7 to +10% change of the total dinner time]. This estimate overlaps with Chen and Rohla’s estimate (19–24 minutes shorter), Frimer and Skitka’s reinterpretation of Chen and Rohla’s data (4–5 minutes shorter), and the null. Including only participants who were confident about their arrival and departure times to within 5 minutes and even to 1 minute did not alter the pattern of results (see Table 2), nor did removing statistical outliers (see S1 Table in S1 File) or adding a quadratic diversity term (see S2 Table in S1 File). Fig 2 show these effect estimates, those of Chen and Rohla [16] as well as Frimer and Skitka [18], and those from Study 2 and the mega-analyses.

**Table 2:** Zero-order effects of political diversity on Thanksgiving dinner duration in 2018.

Predictor of Dinner Duration	All Participants (*N* = 579)				Confidence within 5 mins (*N* = 362)				Confidence within 1 min (*N* = 120)			
	B [95%CI]	β	*p*	*p*_*BIC*_*(H*_*0*_*|D)*	B [95%CI]	β	*p*	*p*_*BIC*_*(H*_*0*_*|D)*	B [95%CI]	β	*p*	*p*_*BIC*_*(H*_*0*_*|D)*
(Constant)	311 [291, 331]		< .001		318 [294, 342]		< .001		326 [275, 376]		< .001	
Diversity	8 [-53, 69]	.011	.797	91%	-56 [-130, 18]	-.079	.137	74%	-10 [-170, 149]	-.012	.896	84%

Given the null finding, and the inferential limits of null hypothesis statistical testing, we used a Bayesian approach to assess the likelihood that the null model was true given the data. We found a 91% chance that the null is true and a 9% chance that the alternative hypothesis is true given the data using the full sample, with substantively similar estimates using tighter inclusion criteria (see Table 2). These results favor the conclusion that diversity is not associated with Thanksgiving dinner duration and fail to replicate Chen and Rohla based on the direction-and-significance criterion.

Contextual factors and potential confounds could have suppressed an effect of diversity on duration in the prior analysis. To test this possibility, we included the overall political leaning of the dinner, dinner location (dummy variables), the start time of the dinner (z-scores), the number of people at the dinner (z-scores), age of the respondent (z-scores), gender (male = 1, else = 0), and race (white = 1, else = 0) in the model. Holding these factors constant, political diversity still did not predict dinner duration, and Bayesian analyses favored the null over the alterative hypothesis by 91% to 9% (see Table 3). Including only those participants who were confident of the times they reported to within 5 minutes and 1 minute respectively had the effect of reducing the sample size substantially, but otherwise not substantively changing the pattern of results. All analyses successfully replicated both Chen and Rohla [16] and Frimer and Skitka [18] in terms of effect overlap but none did in terms of direction and significance (see Tables 4 and 5).

**Table 3:** Conditioned effects of political diversity on Thanksgiving dinner duration in 2018.

Predictor of Dinner Duration	All Participants				Confidence within 5 mins				Confidence within 1 min			
	(*N* = 579)				(*N* = 362)				(*N* = 120)			
	B [95%CI]	β	*p*	*p*_*BIC*_*(H*_*0*_*|D)*	B [95%CI]	β	*p*	*p*_*BIC*_*(H*_*0*_*|D)*	B [95%CI]	β	*p*	*p*_*BIC*_*(H*_*0*_*|D)*
(Constant)	163 [93, 234]		< .001		135 [46, 224]		.003		125 [-116, 366]		.305	
Diversity	16 [-51, 83]	.022	.635	91%	-40 [-122, 42]	-.057	.338	84%	-42 [-239, 154]	-.049	.669	84%
Political Leaning	0 [-23, 24]	.001	.976		0 [-28, 28]	-.001	.989		36 [-32, 103]	.119	.298	
Location = My Place	201 [137, 265]	.620	< .001		226 [147, 304]	.697	< .001		283 [60, 506]	.647	.013	
Location = Someone Else’s	144 [79, 209]	.448	< .001		180 [103, 258]	.571	< .001		232 [15, 449]	.548	.036	
Start Time (z-scores)	-61 [-73, -48]	-.375	< .001		-55 [-71, -40]	-.357	< .001		-55 [-88, -22]	-.303	.001	
# People (z-scores)	6 [-7, 19]	.038	.359		12 [-4, 29]	.076	.140		-6 [-43, 32]	-.028	.766	
Age (z-scores)	-11 [-23, 1]	-.068	.084		-8 [-24, 7]	-.055	.271		-22 [-61, 16]	-.112	.258	
Gender (male = 1, else = 0)	12 [-12, 37]	.038	.329		1 [-29, 31]	.003	.946		-10 [-83, 63]	-.026	.787	
Race (white = 1, else = 0)	-27 [-59, 6]	-.065	.105		-15 [-55, 26]	-.036	.479		-13 [-115, 88]	-.025	.795	

**Table 4:** Did each analysis replicate the corresponding effects from the original study [16] and Frimer and Skitka’s [18] reinterpretation in terms of the estimated effect overlap criterion? Numbers in brackets represent 95% confidence interval estimates of the dinner shortening effect of political diversity (in minutes).

	Sample	Chen & Rohla [16]		Frimer & Skitka [18]	
		Zero Order	Partial	Zero Order	Partial
		[-24, -19]	[-70, -35]	[-5, -4]	[-14, -7]
Study 1 (Trump Approval)	All	yes	no	yes	yes
	5 mins	yes	yes	yes	yes
	1 min	yes	yes	yes	yes
Study 2 (Trump Approval)	All	yes	yes	yes	yes
	5 mins	yes	yes	yes	yes
Study 2 (Impeachment)	All	yes	yes	yes	no
	5 mins	yes	yes	yes	yes
Study 2 (Party)	All	yes	yes	yes	yes
	5 mins	yes	yes	yes	yes
Mega-Analysis	All	yes	yes	yes	yes
**Replication Success Rate**		**100%**	**90%**	**100%**	**90%**

Samples are either all participants or restricted to those that were confident about their reported arrival and departure time to within 5 minutes or 1 minute.

**Table 5:** Did each analysis replicate the corresponding effects from the original study [16] in terms of the direction-and-significance criterion? Samples were either all participants or restricted to those that were confident about their reported arrival and departure time to within 5 minutes or 1 minute.

	Sample	Chen & Rohla [16]	
		Zero Order	Partial
Study 1 (Trump Approval)	All	no	no
	5 mins	no	no
	1 min	no	no
Study 2 (Trump Approval)	All	no	no
	5 mins	no	no
Study 2 (Impeachment)	All	no	yes
	5 mins	no	no
Study 2 (Party)	All	no	yes
	5 mins	no	yes
Mega-Analysis	All	no	yes
**Replication Success Rate**		**0%**	**40%**

Of tangential interest, we note that dinners at home and dinners at other people’s homes were several hours longer than dinners at restaurants, and that the earlier the start time, the longer the dinner. Political leaning did not predict dinner duration, nor did the number of people present, or the age, race, or gender of the respondent.

Next, we tested the proposed and pre-registered moderator, the degree to which the conversation centered on politics. If talking about politics is what makes political diversity strain family ties, then we should expect a diversity × talking politics interaction in predicting dinner duration. This prediction was unsupported (see Table 6). We did find a marginal effect of talking politics such that the more dinners centered on politics, the *longer* they lasted. However, the degree to which the conversation centered on politics did not moderate the effect of diversity on dinner duration. Including only those participants who were confident of the times they reported to within 5 minutes and 1 minute respectively had the effect of reducing the sample size substantially, but otherwise not substantively changing the pattern of results.

**Table 6:** Results from regression analyses testing whether political diversity is associated with shorter Thanksgiving dinners in 2018 and whether the conversation centering on politics made diversity’s effect on dinner duration even more potent.

Predictor of Dinner Duration	All Participants			Confidence within 5 mins			Confidence within 1 min		
	(*N* = 579)			(*N* = 362)			(*N* = 120)		
	B [95%CI]	β	*p*	B [95%CI]	β	*p*	B [95%CI]	β	*p*
(Constant)	310 [289, 330]		< .001	314 [290, 339]		< .001	335 [284, 386]		< .001
Diversity	10 [-51, 72]	.014	.744	-48 [-122, 27]	-.067	.209	-37 [-196, 122]	-.042	.645
Talk Politics (z-scores)	25 [4, 45]	.153	.021	19 [-5, 42]	.128	.121	68 [7, 128]	.365	.028
Diversity × Talk Politics	-28 [-91, 34]	-.059	.375	-7 [-78, 64]	-.017	.841	-126 [-285, 33]	-.257	.120

**Fig 3:**
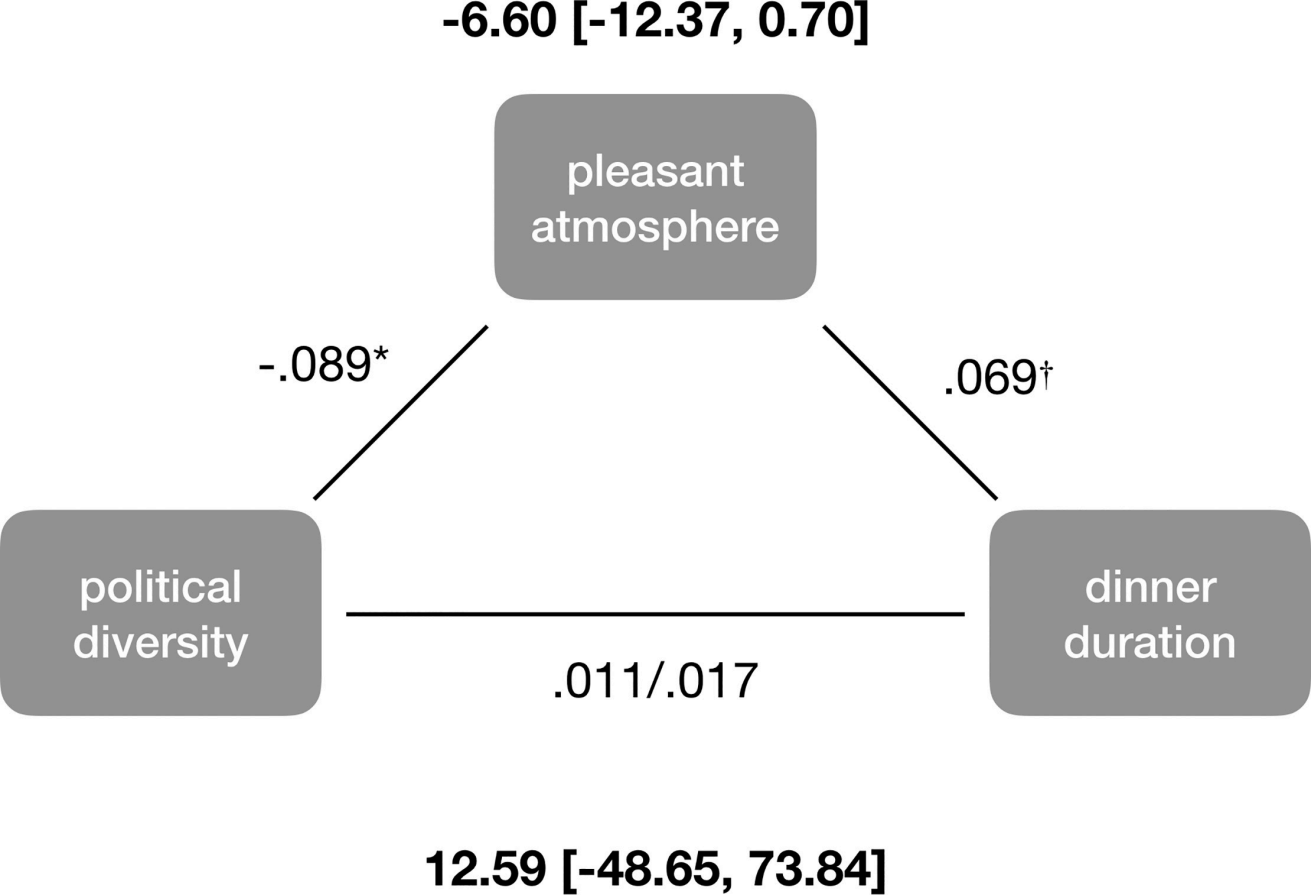
Mediation model assessing whether an unpleasant social atmosphere helped explain why political diversity is associated with shorter Thanksgiving dinners. Direct and indirect effects shown in boldface. † *p* < .10, * *p* < .05.

Finally, using a mediation model, we tested the pre-registered prediction that political diversity would be associated with a shorter dinner duration because diversity reduces how pleasant the social atmosphere is. Fig 3 displays the results of a bias-corrected bootstrapped mediation model. As predicted, political diversity was associated with a negative social atmosphere. And the more positive the social atmosphere, the (marginally) longer the dinner. However, we did not find support for the prediction that the social atmosphere mediated the effect of diversity on dinner duration.

## Discussion

Using a complementary method to that used in previous research, we found little evidence to support the notion that politically diverse dinners were shorter than politically uniform ones, and we found positive support for the conclusion that diverse dinners tend to be of similar duration as politically uniform ones. Bayesian analyses favored the null over alternative hypotheses. These results provisionally support the idea that families are able to set aside their political differences at Thanksgiving dinner, and that political differences are not straining family ties as much as previously thought.

The notion that political differences are straining family ties led us to predict that this would be especially the case when the topic of conversation drifted to politics. This only rarely happened. Politics was the least common topic of conversation (*sports*, *work*, *friends & family*, and *food* were more common topics). But for those dinners that waded into politics, we expected to find political diversity to sharply shorten the dinner. This notion was unsupported, which could mean that talking politics is unpleasant regardless of the attitudes of the conversationalists or that talking politics is not unpleasant enough to shorten a dinner event.

Following from the notion that political diversity is straining family ties, we also predicted the diversity would sour the social atmosphere, and this negative social atmosphere would in turn predict shorter dinners. We almost found evidence to support for this idea, with the confidence interval of the indirect effect just barely overlapping with zero. One possible explanation for why social atmosphere (almost) mediated the (null) effect of diversity on dinner length is that some unobserved feature of the social environment was working in opposition to the dinner-shortening effect of a soured social atmosphere. For example, diversity might make the atmosphere less pleasant while *also* making it more engaging. If people generally like pleasantness and also like engaging atmospheres, it is possible that two competing forces are canceling each other out. In Study 2, we test for this possible suppression effect.

Previously reported effects did not replicate using the direction and significance criterion however they did replicate with respect to the effect overlap criterion, a pattern that points toward the possibility that the effect size estimate that we used to conduct our power analysis was too large and that a small effect of diversity on dinner might exist. Although the sample size of Study 1 was theoretically grounded, empirically derived, and pre-registered, it produced somewhat imprecise estimates. Our zero-order analysis found that politically diverse dinners were somewhere between 23 minutes shorter and 30 minutes longer than politically uniform dinners, a result that cannot test Frimer & Skitka’s [18] estimate relative to the null. Ideally, a sample would be large enough for effect size estimates not overlap with the more than one prediction. To achieve this, we would require a 95% confidence interval of 4 minutes. Extrapolating the power curve observed in the present study (see the S1 File), we estimate that a sample of almost half a million participants would be needed, a sample size that is not feasibly achieved via crowdsourcing.

The (very) large sample size needed to detect the effect proposed by Chen and Rohla highlights the value of Chen and Rohla’s large data set, which included the smart phone location data of ~10 million Americans. At the same time, it highlights how small in statistical terms the effect they observed was. Our effect size estimate of *r* ~ .01, 95% confidence interval [-.07, .09] is a small fraction (4%) of the size of the effect that is said to be driving it (selective exposure motivation, *r* ~ .30). It might well be the case that political diversity shortens Thanksgiving dinners. But, based on the available evidence, it seems unlikely that the effect is an appreciable one.

Several methodological limitations of Study 1 are also worth noting. First, when asking about who attended dinner and about their political beliefs, the survey asked about people. Some of the named individuals might have been children without clear (or any) political beliefs. In Study 2, we ask about adults specifically. Second, in Study 1, we measured attitudes only with respect to approval or disapproval of President Trump. Although Trump is a defining figure in contemporary American politics, it would be helpful to include other measures of political attitudes to test whether the effects observed generalize.

## Study 2: Thanksgiving 2019

Pre-registered (https://osf.io/3se4z/registrations) Study 2 again tested whether politically diverse Thanksgiving dinners were shorter than politically uniform ones, only this time in the year 2019. We again examined whether the dinner-shortening effect of political diversity was especially strong when the conversation gravitated to politics, and whether diversity soured the social atmosphere, which in turn reduced the dinner duration.

There were five changes from Study 1 to Study 2. First, we included a second potential mediator—the degree to which the conversation was engaging. This inclusion allowed us to test a suppression model whereby diversity makes dinner less pleasant but at the same time more engaging, and these two forces cancel out one another in terms of the effect on the overall length of the dinner. Second, in Study 2 we asked about “adults” at dinner, rather than about “people”, to increase the likelihood that those named had political views. Third, we expanded our measurement of diversity to measure it three ways, with respect to attitudes toward President Trump, attitudes toward the impeachment of President Trump (the House of Representatives passed Articles of Impeachment 3 weeks after this study), and party preference. Fourth, we added questions about how far and for how long participants traveled to reach the Thanksgiving event so that we could control for the effect of traveling on dinner duration (as Chen and Rohla did). And fifth, we doubled the sample size to raise the likelihood of rejecting the null and detecting small effects.

### Method

#### Sample

We decided to double the sample size from Study 1 to redouble our likelihood of detecting an effect sourced in selective exposure motivation. Our pre-registered goal was to recruit 2000 participants in Part I with hopes of recruiting 1000 for Part II. Our final sample was *N* = 1146, which gave us 95% power to detect effects of |*r*| > .106 and 80% power to detect |*r*| > .083.

#### Procedure

This study was reviewed and approved by the University Human Research Ethics Board at the University of Winnipeg (HE12152). Part I was posted on Amazon’s Mechanical Turk on November 26, 2019 two days before Thanksgiving Day 2019 (November 28). In Part I, payment was $0.10. Participants reported if and when they planned on having Thanksgiving Dinner, received instructions to take note of the *precise* times that they arrive and leave, and then to complete Part II the day after Thanksgiving. We invited those who reported that they would have Thanksgiving Dinner on Thursday, November 28 to complete Part II using TurkPrime. We limited our sample to Thursday dinners so that we could launch part II the morning after Thanksgiving (Friday morning) so that memories would still be fresh. With 91% of dinners occurring on the Thursday (see below), we had more than enough participants from Part I to fill our Part II sample. As indicated in our pre-registered plan, we stopped recruiting Part II participants once we had 1000 participants. This left us with 1148 respondents. We excluded 2 participants who in Part II reported that they did not have Thanksgiving dinner after all, leaving *N* = 1146.

In Part II, payment was $1.00. Participants reported where they had Thanksgiving dinner, how far they traveled to get there, and how long their travels took. Next, they reported their arrival and departure times, and described the topic of conversation and the social atmosphere. They then reported how many people were at Thanksgiving Dinner, and each of those individuals’ attitudes toward President Trump, their attitudes toward the impeachment of President Trump, and their partisan leaning. Finally, they supplied demographic information and receive a full debriefing.

*Part I*. A question asked, “Do you plan on having Thanksgiving Dinner with other people this year?” Response options were *yes* and *no*. Most (93%) responded in the affirmative. Only those participants that selected yes were then asked, “When?” with response options being *Thurs Nov 28*, *Fri Nov 29*, *Sat Nov 30*, *Sun Dec 1*, and *other*. The most common date was the Thursday (91%), with 3% each having dinner on the Friday and Saturday, 2% having the dinner on Sunday and 1% indicating some other date. Only those participants that indicated that they were having Thanksgiving dinner and that it would be on Thursday qualified for Part II. They then read the same message as in Study 1.

*Part II*. In Part II, participants were asked about the dinner location, their travels to dinner, the event’s duration, the topic of conversation, the social atmosphere, and the attendees.

*Dinner location*. The question asked, “where did you have Thanksgiving dinner?” Response options were: *I didn’t have Thanksgiving dinner* (0.2%), *at my place* (47.9%), *at a restaurant* (3.6%), and *at someone else’s place* (48.3%). The final option allowed participants to specify where in a text box (not analyzed). We excluded both participants who indicated that they did not have Thanksgiving dinner after all in all subsequent analyses.

*Travel to dinner*. A first question asked about travel distance. The question was, “Approximately how far (in miles) did you travel to get to Thanksgiving dinner?” Response options were 0, 1, 2, 5, 10, 20, 50, 100, 200, 500, 1000, 2000, 5000, and 10000 miles. The raw distances (see S3 Table in S1 File) yielded a skewed distribution (*skew* = 12.72) so we converted 0 miles to 0.1 miles (to retain all data in its ordinal form in the next step) and log-transformed the distance (*M* = 0.128, *SD* = 1.143), which reduced the skewness to an acceptable 0.31. We then computed z-scores for analyses. The next question asked about how long it took to get there. The question asked, “How long did it take to get to Thanksgiving Dinner?” Participants responded using two dropdown menus. The first listed the number of hours (0, 1, 2, …, 22, 23, 24 hours) and the second listed the number of minutes (0, 5, 10, …, 45, 50, 55 minutes). S4 Table in S1 File displays the frequencies, which formed a skewed (17.06) distribution. We thus performed the same log transformation to reduce the skew (-0.19) before computing z-scores.

*Thanksgiving dinner duration*. We asked participants to report the time they arrived and departed from Thanksgiving Dinner. The arrival question asked, “What time did you arrive at Thanksgiving dinner? If you had dinner at home, what time did the first guest arrive?” They reported the time on two dropdown menus. The first listed the hour (*12am*, *1am*, *2am*, …, *9pm*, *10pm*, *11pm*) and the second listed the minute (:*00*,: *05*,: *10*, …,: *45*,: *50*,: *55*). The departure question was similar. We then multiplied the hour by 60 (e.g., *2am* = 120, *1pm* = 780) and added the number of minutes (e.g.,: *35* = 35) to derive arrival and departure times (see S4 Fig in S1 File).

After reporting a time, they reported their confidence of their time by completing the statement, “I feel confident that this time is…” Response options were *within 1 minute of when I actually arrived* (22.1%), *within 5 minutes of when I actually arrived* (32.0%), *within 10 minutes…* (26.0%), *within 20 minutes…* (10.5%), *within 30 minutes…* (6.5%), *within 1 hour…* (1.9%), and *within 4 hours…* (1.0%). They were then asked analogous questions about the time they left Thanksgiving dinner. We counted the least accurate confidence estimate to be each person’s confidence estimate (reported above in parentheses). And we computed the duration of Thanksgiving dinner by subtraction (*M* = 382 minutes, *SD* = 260 minutes, range: 16–1440 minutes). Dinner duration was normally distributed, skew = 1.78 (see S5 Fig in S1 File for a histogram).

*Topic of conversation*. Participants reported the topic of conversation. A question asked, “How often did the conversation center on…?” For each of *politics*, *sports*, *work*, *friends & family*, and *food*, participants responded on a 101-point scale anchored at 0 (*rarely*), 33 (*once in a while*), 67 (*often*), and 100 (*constantly*). In descending order, the most common topics of conversation were *family and friends* (*M* = 63, *SD* = 23), *food* (*M* = 59, *SD* = 25), *work* (*M* = 29, *SD* = 23), *sports* (*M* = 29, *SD* = 28), and *politics* (*M* = 14, *SD* = 18).

*Social atmosphere*. Participants described the social atmosphere by responding to the question, “How was the social atmosphere at dinner?” There were 12 items, which tapped how pleasant (α = .83) and engaging (α = .75) the atmosphere was, respectively. The *pleasant* items were the same as they were in Study 1 (items: *pleasant*, *happy*, *relaxed*, *tense*, *sad*, and *offensive*, with the final three being reverse scored). We introduced the (pre-registered) *engaging* scale in Study 2 (items: *engaging*, *interesting*, *educational*, *boring*, *dull*, and *forgettable* with the final three being reverse scored). Responses were on a 101-point scale, anchored at 0 (*not at all*), 33 (*slightly*), 67 (*somewhat*), and 100 (*very*). Overall, the social atmosphere was pleasant (*M* = 84, *SD* = 14) and engaging (*M* = 66, *SD* = 16).

*Political diversity*. Unlike in Study 1, which measured political diversity only with respect to attitudes toward President Trump, we measured political diversity in three ways in Study 2. First, we measured political diversity as we did in Study 1. The only difference in Study 2 was that we now asked about “adults”, rather than about “people”. The average dinner had 7.17 attendees (*SD* = 2.71). Just 2.5% of Thanksgiving dinners had 2 attendees. On average, participants reported the attitudes of 6.87 attendees, including themselves (*SD* = 2.64). The average Trump approval diversity score was .24 (*SD* = .22). Second, we measured political diversity by asking about whether or not President Trump should be impeached. The question asked, “Does {name} think that President Trump should be impeached?” with an attendee’s name replacing {name} in each instance. Response options were *no* (*M*_*attendees*_ = 1.88, *SD* = 2.36), *neutral* (*M* = 1.04, *SD* = 1.67), *yes* (*M* = 2.67, *SD* = 2.37), and *I don’t know* (*M* = 0.00, *SD* = 0.00). We then computed diversity scores using the same approach as for approval of Trump. The average Trump impeachment diversity score was .30 (*SD* = .24). For both Trump attitudes and Trump impeachment stances, we also computed overall political leaning of the dinner scores, with higher scores being more pro-Trump, and computed in the same way as in Study 1.

Third, we measured political diversity by asking about party identification. The question asked, “How does {name} lean politically?” Responses were on a 7-point scale anchored at -3 (*strongly Democratic*), -2 (*moderately Democratic*), -1 (*slightly Democratic*), 0 (*neutral/unknown*), 1 (*slightly Republican*), 2 (*moderately Republican*), and 3 (*strongly Republican*). The average political leaning was centrist (*M* = -0.29, *SD* = 1.88). Our pre-registered operationalization of political diversity was the *SD* of the leanings divided by the constant 4.2426. We used this particular constant to scale the partisan diversity variable to have the same possible range as the other two diversity scores (4.2426 = the *SD* of the most diverse possibility; -3 and 3). The average partisan diversity score was .29 (*SD* = .17). We also computed overall partisan leaning scores, with higher scores being more Republican, computed as the average partisan leaning, divided by 3 (so that scores could range from -1 to 1). The three measures of diversity correlated with one another (all *p*s < .001) in the range of *r* = .35 to .59, whereas the three measure of political leaning correlated with one another more strongly, ranging from *r* = .71 to .79.

### Results

**Fig 4:**
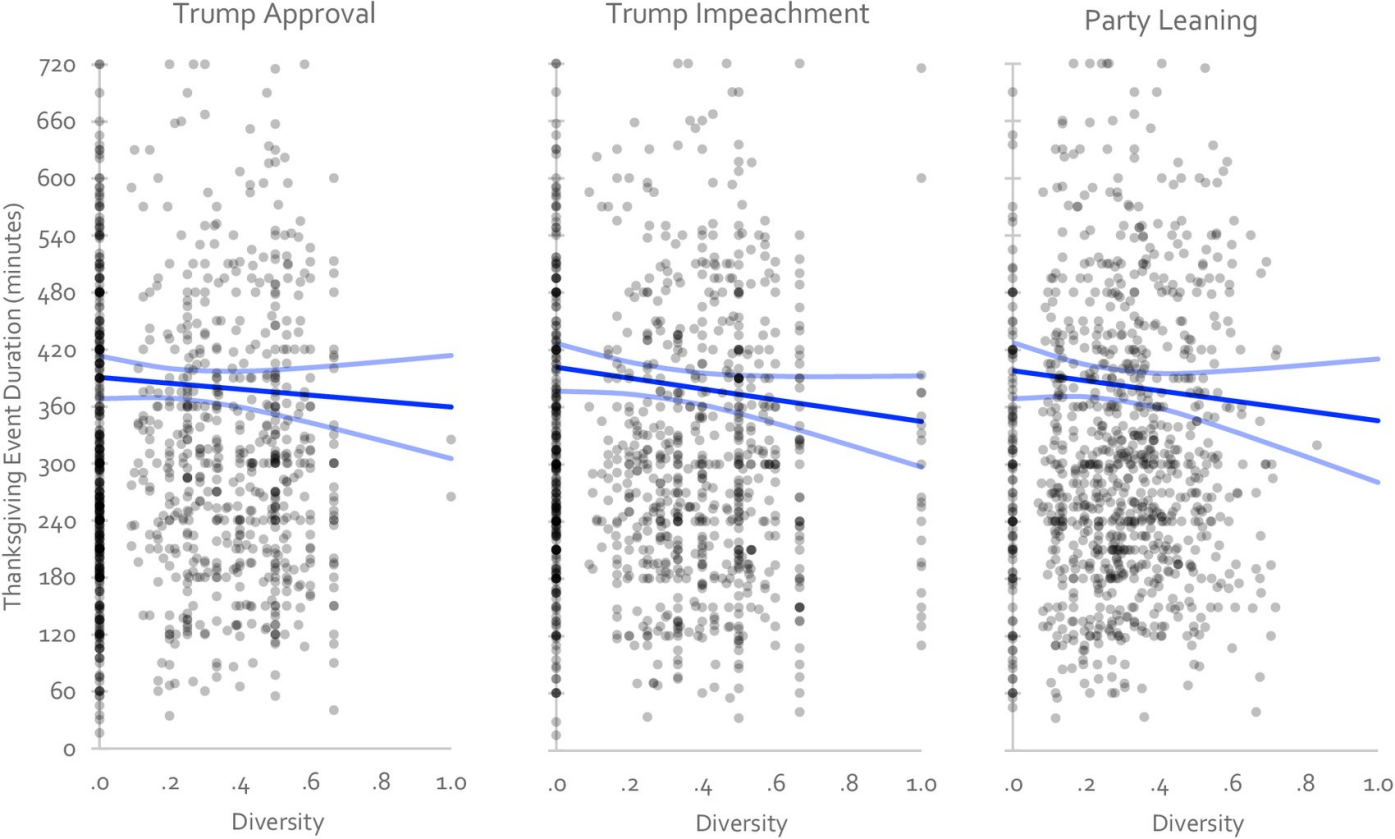
Scatterplot and OLS regression line (with 95% confidence interval) showing the relationship between political diversity and Thanksgiving dinner duration in 2019. Each dot represents a Thanksgiving Dinner event.

We first estimated the zero-order effect of diversity on Thanksgiving dinner duration with all participants included by testing whether diversity predicts duration in an OLS regression analysis. Across the three operationalizations of political diversity, we failed to reject the null hypothesis that diversity does not predict dinner duration. Following our pre-registered interpretive strategy (see Study 1), we found that when politically diversity was defined in relation to approval of President Trump, diverse dinners were 14 minutes (4%) shorter, 95%CI = [17 mins longer, 45 mins shorter] than uniform ones. When politically diversity was defined in relation to the impeachment of President Trump, diverse dinners were 26 minutes (7%) shorter, 95%CI = [4 mins longer, 55 mins shorter] than uniform ones. And when politically diversity was defined in relation to partisan leaning, diverse dinners were 24 minutes (7%) shorter, 95%CI = [16 mins longer, 63 mins shorter] than uniform ones (see Fig 4 and Table 7). Including only those participants that claimed that their reported times were within 5 minutes of the actual time yielded a similar pattern of results, as did removing statistical outliers (see S1 Table in S1 File) or adding a quadratic diversity term (see S2 Table in S1 File). Like in Study 1, all three estimates’ confidence intervals overlapped with the null, but also with Chen and Rohla’s claims, as well as with Frimer and Skitka’s reinterpretation of the same, meaning that replication by the effect overlap criterion was uniformly successful (see Table 4). In contrast, none of the previously reported effects replicated by the direction and significance criterion (see Table 5).

**Table 7:** Zero-order effects of political diversity on Thanksgiving dinner duration in 2019. Diversity was operationalized threefold, as attitudes toward Trump, attitudes toward impeachment, and party preferences. Models included either all participants, participants that reported being confident about the times they reported to within 5 minutes of the actual time.

		All (*N* = 1146)				Within 5 mins confidence (*N* = 617)			
	Predictor	B [95%CI]	β	*p*	*p*_*BIC*_*(H*_*0*_*|D)*	B [95%CI]	β	*p*	*p*_*BIC*_*(H*_*0*_*|D)*
**Trump**									
	(Constant)	391 [368, 413]		< .001		386 [355, 416]		< .001	
	Diversity	-31 [-100, 37]	-.027	.370	91%	-48 [-140, 43]	-.043	.300	86%
**Impeachment**									
	(Constant)	402 [377, 427]		< .001		403 [369, 437]		0	
	Diversity	-57 [-122, 8]	-.052	.085	77%	-91 [-179, -2]	-.083	.045	58%
**Party**									
	(Constant)	398 [369, 428]		< .001		377 [336, 418]		< .001	
	Diversity	-52 [-140, 36]	-.035	.243	88%	-16 [-136, 103]	-.011	.786	91%

Given the null findings, we used a Bayesian approach to assess the likelihood that the null model was true given the data. We found that the null model had a 77%-91% likelihood of being true in the full sample and a 58%-91% chance of being true among participants who were confident about their reported times to within 5 minutes (see Table 7). These results again favor the conclusion that Thanksgiving dinner duration probably does not covary with political diversity.

Contextual factors and possible confounds could have suppressed an effect of diversity on duration in the prior analysis. To test this possibility, we included the overall political leaning of the dinner, dinner location (dummy variables), travel distance (z-scores), travel time (z-scores), the start time of the dinner (z-scores), the number of people at the dinner (z-scores), age of the respondent (z-scores), gender, and race in the model. Holding these factors constant yielded mixed results. Political diversity predicted shorter dinners in two of six tests. There were marginal effects in two of six and null effects in the final two of six. The significant effects were found when diversity was operationalized with regards to partisan affiliation, and not when defined with respect to President Trump. Given these mixed findings, we used a Bayesian approach to assess the likelihood that the null model was true given the data. When diversity was defined with respect to President Trump, we found that the null model had a 65%-83% likelihood of being true (depending on the operationalization of diversity and inclusion criteria). In contrast, when diversity was defined with respect to partisan leaning, the null model had a 23%-45% chance of being true (see Table 8). Replication success rate was 100% for Chen and Rohla and 83% for Frimer and Skitka using the effect size overlap criterion (see Table 4), but only 50% using the direction and significance criterion (see Table 5).

**Table 8:** Conditioned effects of political diversity on Thanksgiving dinner duration in 2018.

	Predictor	All (*N* = 1146)				Within 5 mins confidence (*N* = 617)			
		B [95%CI]	β	*p*	*p*_*BIC*_*(H*_*0*_*|D)*	B [95%CI]	β	*p*	*p*_*BIC*_*(H*_*0*_*|D)*
Trump									
	(Constant)	291 [206, 376]		< .001		257 [143, 371]		< .001	
	Diversity	-54 [-120, 11]	-.047	.106	80%	-77 [-161, 7]	-.068	.073	67%
	Political Leaning	-20 [-42, 1]	-.053	.068		-23 [-50, 5]	-.063	.103	
	Location = My Place	129 [41, 218]	.248	.004		162 [40, 284]	.312	.009	
	Location = Someone Else’s	108 [30, 187]	.208	.007		138 [36, 241]	.269	.008	
	Travel Distance (z-scores)	-8 [-38, 22]	-.030	.611		-15 [-59, 29]	-.057	.502	
	Travel Time (z-scores)	11 [-9, 31]	.042	.291		13 [-16, 42]	.047	.380	
	Start Time (z-scores)	-104 [-119, -90]	-.395	< .001		-118 [-138, -98]	-.436	< .001	
	# People (z-scores)	21 [5, 36]	.076	.008		29 [9, 48]	.108	.004	
	Age (z-scores)	-5 [-20, 9]	-.021	.481		-17 [-36, 2]	-.068	.075	
	Gender (male = 1, else = 0)	32 [3, 61]	.060	.030		52 [15, 90]	.100	.006	
	Race (white = 1, else = 0)	-40 [-76, -3]	-.064	.032		-49 [-99, 1]	-.077	.056	
Impeachment									
	(Constant)	269 [180, 358]		< .001		256 [138, 374]		< .001	
	Diversity	-49 [-115, 17]	-.045	.143	83%	-81 [-169, 6]	-.075	.069	65%
	Political Leaning	-21 [-46, 4]	-.052	.099		-15 [-46, 17]	-.037	.369	
	Location = My Place	149 [59, 239]	.288	.001		174 [48, 299]	.332	.007	
	Location = Someone Else’s	140 [60, 220]	.271	.001		155 [50, 261]	.301	.004	
	Travel Distance (z-scores)	-11 [-41, 20]	-.041	.491		-15 [-60, 30]	-.055	.519	
	Travel Time (z-scores)	7 [-13, 27]	.028	.487		12 [-18, 42]	.044	.422	
	Start Time (z-scores)	-104 [-119, -89]	-.399	< .001		-118 [-138, -98]	-.433	< .001	
	# People (z-scores)	13 [-2, 29]	.048	.095		23 [2, 43]	.082	.029	
	Age (z-scores)	-4 [-19, 11]	-.016	.585		-17 [-36, 2]	-.067	.083	
	Gender (male = 1, else = 0)	36 [7, 65]	.069	.015		59 [21, 97]	.113	.003	
	Race (white = 1, else = 0)	-41 [-77, -5]	-.067	.027		-57 [-107, -7]	-.090	.026	
Party									
	(Constant)	300 [216, 384]		< .001		262 [146, 378]		< .001	
	Diversity	-118 [-202, -35]	-.078	.006	23%	-128 [-239, -17]	-.085	.024	45%
	Political Leaning	-12 [-44, 19]	-.022	.437		-25 [-66, 16]	-.045	.231	
	Location = My Place	157 [69, 244]	.300	< .001		183 [60, 305]	.354	.003	
	Location = Someone Else’s	123 [46, 199]	.235	.002		150 [47, 253]	.294	.005	
	Travel Distance (z-scores)	-3 [-32, 27]	-.010	.868		-10 [-54, 34]	-.037	.663	
	Travel Time (z-scores)	8 [-12, 28]	.031	.424		11 [-18, 41]	.042	.445	
	Start Time (z-scores)	-104 [-118, -90]	-.395	< .001		-113 [-133, -93]	-.421	< .001	
	# People (z-scores)	14 [-1, 29]	.054	.065		19 [0, 39]	.075	.053	
	Age (z-scores)	-7 [-22, 7]	-.029	.319		-17 [-36, 2]	-.069	.073	
	Gender (male = 1, else = 0)	31 [3, 60]	.059	.031		54 [17, 92]	.105	.005	
	Race (white = 1, else = 0)	-42 [-78, -6]	-.068	.023		-45 [-94, 5]	-.071	.075	

These analyses also found that dinners at home and dinners at other people’s homes were several hours longer than dinners at restaurants; the earlier the start time, the longer the dinner; dinners with more people were longer than dinners with fewer people; males reported longer dinners than females; and white people reported shorter dinners than non-white people. Political leaning did not predict dinner duration, nor did the age of the respondent, the travel time or distance to the dinner.

Next, we tested the proposed and pre-registered moderator, the degree to which the conversation centered on politics. If talking about politics is what makes political diversity so toxic to family ties, then we should expect a diversity × talking politics interaction in predicting dinner duration. This prediction was unsupported in five of six tests and marginally significant in the sixth (Table 9). We also found a marginal effect of talking politics in one of six tests such that talking politics was associated with a longer dinner duration. (Using residual dinner durations from the full sample and party leaning yielded the same pattern of results.)

**Table 9:** Results from regression analyses testing whether political diversity is associated with shorter Thanksgiving dinners in 2019 and whether the conversation centering on politics made diversity’s effect on dinner duration even more potent.

		All (*N* = 1146)			Within 5 mins confidence (*N* = 617)		
		B [95%CI]	β	*p*	B [95%CI]	β	*p*
Trump							
	(Constant)	390 [368, 413]		< .001	385 [354, 416]		< .001
	Talk Politics	1 [-21, 23]	.005	.909	19 [-14, 52]	.070	.259
	Diversity	-34 [-104, 35]	-.030	.330	-48 [-140, 44]	-.043	.306
	Talk Politics × Diversity	-35 [-103, 34]	-.043	.321	-48 [-144, 48]	-.061	.327
Impeachment							
	(Constant)	401 [376, 426]		< .001	403 [368, 437]		< .001
	Talk Politics	-12 [-36, 13]	-.046	.349	-10 [-48, 28]	-.037	.604
	Diversity	-55 [-121, 11]	-.050	.103	-92 [-183, -1]	-.083	.046
	Talk Politics × Diversity	13 [-53, 79]	.019	.697	56 [-40, 152]	.081	.255
Party							
	(Constant)	398 [368, 427]		< .001	375 [334, 416]		< .001
	Talk Politics	9 [-18, 36]	.035	.514	36 [-4, 76]	.132	.080
	Diversity	-53 [-142, 36]	-.035	.243	-9 [-130, 113]	-.006	.889
	Talk Politics × Diversity	-54 [-137, 28]	-.068	.197	-105 [-222, 11]	-.134	.077

**Fig 5:**
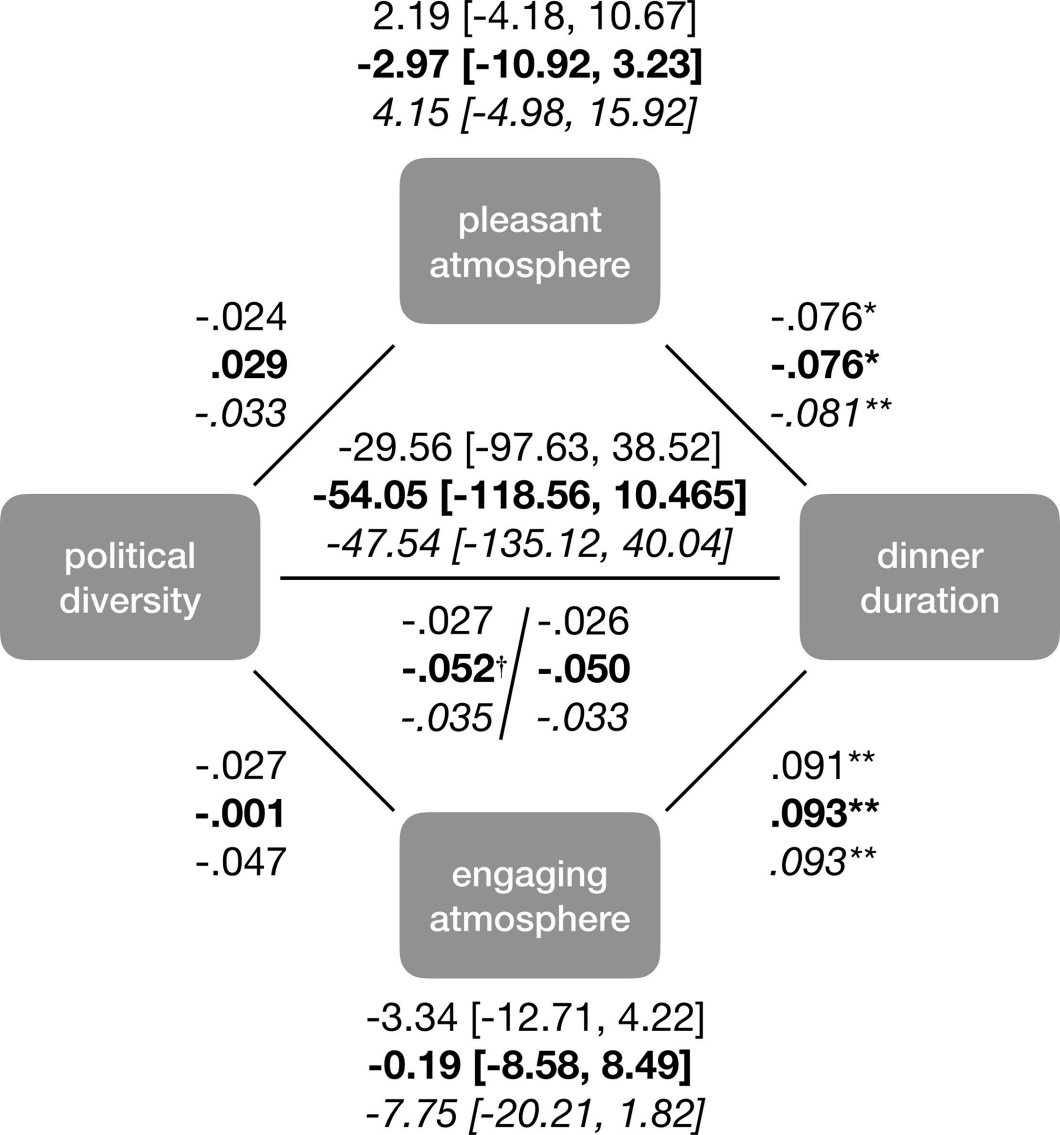
Mediation model assessing whether an unpleasant and/or engaging social atmosphere helped explain why political diversity might not be associated with shorter Thanksgiving dinners. Numbers in plain text represent diversity defined in terms of attitudes toward Trump. Numbers in boldface represent diversity defined in terms of attitudes toward impeachment. Numbers in italics represent diversity defined in terms of party affiliation. † *p* < .10, * *p* < .05, ** p < .01.

Finally, using a mediation model, we tested the pre-registered suppression model—that political diversity would not be associated with dinner duration because diversity both makes the atmosphere more pleasant (shrinking the evening) but also more engaging (lengthening the evening). Fig 5 displays the results. The model was not supported. Political diversity was associated with neither a pleasant atmosphere nor with an engaging one. However, the more engaging and less pleasant the social atmosphere, the longer the dinner. In the S1 File, we tested whether this model might yield different conclusions when examining the positive result found in Table 8, that diversity (defined with respect to partisan affiliation) predicts dinner duration while controlling for various contextual factors. In this model, instead of using the raw dinner durations, we used the residual dinner durations after removing the effects of dinner location, race, etc. Aside from a significant indirect and direct effect of diversity on duration, the model remained unchanged, meaning that the mediation model was unsupported (see S6 Fig in S1 File).

## Discussion

Despite doubling our sample size, Study 2 still failed to produce consistent evidence that political diversity predicted shorter Thanksgiving dinners. Our estimates had very wide confidence interval estimates, which overlapped with both Chen and Rohla’s findings and with Frimer & Skitka’s reinterpretation, meaning that effect overlap replication success was high. However, most Bayesian analyses favored the null over these alternative hypotheses. These results again support the idea that political differences are not straining family ties as much as previously thought. Like in Study 1, we again failed to find evidence of moderation and mediation derived from the notion that political diversity is straining family ties because people dislike hearing from the other side of the Culture War. That said, doubling the sample size increased the replication rate using the direction-and-significance criterion from 0% to 50%, which again points to the possibility that a very small effect still exists. With effect size estimates ranging from *r* = -.01 to -.09 depending on the operationalization of political diversity, the available evidence suggests that the effect of diversity on dinner duration is unlikely to be an appreciable one.

Study 2 allayed several minor methodological concerns raised in Study 1. The inclusion of the degree to which the conversation was engaging mediator, asking about adults rather than about people, and asking about travel time and duration turned out to yield few new insights. However, including three measures of diversity—operationalized with respect to attitudes toward Trump, attitudes toward impeachment, and partisan leaning—did pay dividends. Specifically, when diversity was defined with respect to partisan leaning (but not with respect to President Trump), we found some evidence that diversity predicts a shorter dinner duration when controlling for a raft of contextual factors. It could be that only when diversity is measured with respect to partisan leaning and/or using a larger, more sensitive scale (7-point in this case), and when controlling for contextual factors, does diversity predict dinner duration. Alternatively, this finding could be a one-off (that is, a false positive). Future research should investigate these possibilities. Although not conclusive, the present findings tended to support the null over the claim that diversity predicts Thanksgiving dinner duration.

## Mega-analysis

To maximize the precision of our estimate of the effect of diversity on dinner duration, we conducted a mega-analysis with all data from Studies 1 and 2 (*N* = 4013), which delivered 95% power to detect |*r*|s > .057 and 80% power to detect |*r*|s > .044. We tested whether diversity predicted dinner duration in a zero-order OLS regression and again failed to reject the null hypothesis (see Table 10). Following our preregistered interpretative strategy, we found that dinners that were relatively diverse (*M*_diversity_ + 1*SD*_diversity_ = .48) were 11 minutes (3%) shorter on average than dinners that were relatively uniform (*M*_diversity_ - 1*SD*_diversity_ = .04), 95%CI = [27 mins shorter (1%), 4 mins longer (7%)]. These effect estimates replicate Chen and Rohla’s [16] and Frimer and Skitka’s [18] estimates while failing to replicate them using the direction and significance criterion.

**Table 10:** Mega-analytic zero-order and conditioned effects of political diversity on Thanksgiving dinner duration in 2018 and 2019.

	Zero Order Correlation				Conditioned Correlations			
	B [95%CI]	β	*p*	*p*_*BIC*_*(H*_*0*_*|D)*	B [95%CI]	β	*p*	*p*_*BIC*_*(H*_*0*_*|D)*
(Constant)	379 [367, 391]		< .001		292 [250, 335]		< .001	
Diversity	-26 [-62, 10]	-.023	.154	91%	-54 [-89, -20]	-.047	.002	35%
Political Leaning					-11 [-24, 1]	-.027	.077	
Location = My Place					145 [106, 183]	.290	< .001	
Location = Someone Else’s					116 [78, 155]	.233	< .001	
Start Time (z-scores)					-96 [-103, -89]	-.384	< .001	
# People (z-scores)					16 [9, 24]	.065	< .001	
Age (z-scores)					-6 [-13, 1]	-.024	.106	
Gender (male = 1, else = 0)					27 [13, 42]	.054	< .001	
Race (white = 1, else = 0)					-46 [-64, -28]	-.079	< .001	
Diversity Operationalization = Trump Approval					-21 [-38, -4]	-.042	.016	
Diversity Operationalization = Impeachment					0 [-19, 18]	-.001	.962	

When examining conditioned effects, however, we found the diverse dinners were 24 minutes shorter than uniform ones, 95%CI = [9 (2%), 39 (10%)], an effect that reached significance at the .002 level (see Table 10). This estimate successful replicated both Chen and Rohla [16] and Frimer and Skitka [18] by both criteria. That said, a Bayesian analysis only weakly favored the alternative over the null by a margin of 65% to 35%. The effect size estimate was *r*_p_ = -.047, 95%CI = [-.017, .077].

Neither the proposed moderator of talking about politics (see Table 11) nor the proposed mediator (unpleasant atmosphere) were supported in either the zero-order or the conditioned model. To test the conditioned model, we extracted unstandardized residuals of diversity from an analysis in which all of the covariates in Table 10 predicted diversity. The indirect path of an unpleasant atmosphere did not reach significance in either the zero-order model, B = -0.09, 95%CI = [-1.08, 0.69], or in the conditioned model, B = -0.20, 95%CI = [-1.45, 0.58].

**Table 11:** Results from regression analyses testing whether political diversity is associated with shorter Thanksgiving dinners a mega-anaysis and whether the conversation centering on politics made diversity’s effect on dinner duration even more potent.

	Zero-Order Model			Conditioned Model		
	B [95%CI]	β	*p*	B [95%CI]	β	*p*
(Constant)	379 [366, 391]		< .001	372 [364, 380]		< .001
Talk Politics	-1 [-13, 11]	-.002	.926	-6 [-13, 2]	-.022	.168
Diversity	-26 [-62, 10]	-.023	.153	-51 [-90, -13]	-.042	.009
Talk Politics × Diversity	-18 [-54, 18]	-.024	.324	-34 [-72, 4]	-.028	.076

The partial model used diversity residuals after removing the effect of the covariates listed in Table 10.

## General discussion

Are there safe havens from the socially detrimental effects of the Culture War between liberals and conservatives? Previous research [16] found that even Thanksgiving dinner was not immune to the effects of the Culture War, implying that there may be few safe havens from the conflict. Noting limitations in the previous analysis, we conducted two studies aimed to test whether diverse dinners are shorter than uniform ones and to conceptually replicate and extend the original study. Using a complementary method, as well as pre-registered and well-powered studies, we generally failed to reject the null hypothesis (thus failing to replicate Chen and Rohla by the direct-and-significance criterion), and Bayesian analyses tended to favor the null hypothesis that diversity and Thanksgiving dinner duration are unrelated. However, most effect size estimates overlapped with those in the original, leaving open the possibility that a statistically small effect exists. A mega-analysis of zero-order effects again favored the null. However, controlling for the effect of covariates, a mega-analysis suggested that diverse dinners are 24 minutes shorter than uniform ones, 95%CI = [9, 39], representing a 2%-10% reduction in overall dinner time. This result replicates Chen and Rohla’s [16] conclusions by both criteria. The effect size estimate was *r*_p_ = -.047, 95%CI = [-.017, .077], which is approximately six times smaller than that of the proposed explanation (selective exposure). A Bayesian analyses weakly supported the alternative hypothesis by 65% to 35% probability.

For a replication to be meaningful, theoretically relevant contextual factors need to match those of the original. One possible meaningful difference is change over time. The original study [16] was in 2015 and 2016 whereas the present studies were in 2018 and 2019. If tensions between the two parties had calmed over time, then we might expect political diversity to strain family ties less over time. However, polling data [22] suggest that the rift between the political left and right in the U.S. is not in decline; if anything, it is increasing. Another possibility is that election years bring politics to mind for many Americans, and thus amplifies the family-straining effect of differences of opinion. The original study included a Thanksgiving dinner during a presidential election year (2016) whereas the replication studies did not. However, we note that the effects in the original were also found in 2015, which is at the same time in the electoral cycle as 2019. Moreover, the 2019 study took place in the midst of a highly publicized and politically divisive impeachment of a president, and the 2018 study took place in a midterm election year. These considerations suggest that the context in the replication studies were probably germane to the original claims.

A significant difference between the original and the present investigations concern methodology and sample sizes. Whereas the original studies used location data from ~10 million Americans’ smartphones and publicly available election results to measure political diversity and dinner duration, we recruited the self-reports of ~1,500 crowdsourced participants. The sample size differences are substantial and allowed the prior analysis to make more precise estimates and potentially to detect a signal in a (literally and statistically) noisy situation. However, our Bayesian analyses were not equivocal about the null vis-à-vis the alternative. That is, our individual samples were sufficiently large to reach a clear conclusion that politically diverse dinners are no longer or shorter than politically uniform ones, on average. However, our mega-analytic analysis that controlled for covariates favored the opposite conclusion, that diverse dinners are shorter than uniform ones (albeit tentatively so).

The methodological differences between the prior and current analyses are substantial, rendering the present effort a conceptual and not a direct replication of the original. Motivating the change in methodology was noted limitations of the original methodology. However, the present methodology was not without its own limitations. For instance, we relied on participants to report the time that they arrived at and departed from the dinner the next day. Before the dinner, we instructed them to record the precise time of arrival and departure. However, only ~60% of participants reported that they were confident about their reported times to within 5-minutes. Including only these participants yielded similar conclusions. However, it remains a possibility that our methodology for measuring the duration of the dinner introduced a sufficient amount of noise to drown out a real signal.

Noting the methodological limitations of both the prior and the current approaches to measuring political diversity and Thanksgiving dinner warrants some appreciation of the inherent challenges in studying statistically small effects in private settings. Recognizing these challenges temper our objective to the goal of adding useful information to this important question, rather than to offer the final word. That said, we believe that the bulk of evidence thus far suggests that although people expect conversations with unlike-minded others to be painful [5], they over-estimate the severity of the negative affect of these actual conversations [23]. It appears that the Culture War division that exists is not as strong and toxic as generally thought [24], that many norms surrounding civility and politeness remain intact [25, 26]. With perhaps only a small disruption attributed to politics, Americans appear to be largely successful at putting aside their political differences and enjoying Thanksgiving dinner with relatives and friends with whom their differ.

## Supporting information

S1 File (DOCX)
