# Supplementary material for: Are politically diverse Thanksgiving dinners shorter than politically uniform ones?
Source: PLoS One. 2020 Oct 27;15(10):e0239988. doi: 10.1371/journal.pone.0239988 (PMC7591091; doi:10.1371/journal.pone.0239988)
Supplement: S1 File — (DOCX) [file pone.0239988.s001.docx]

Study 1

**Figure S1**. Histogram of Thanksgiving 2018 dinner arrival and departure times.

**Figure S2**. Histogram of Thanksgiving 2018 dinner durations.

**Power curve**

The two closest predictions about the effect of political diversity on Thanksgiving dinner duration are 0 minutes (the null) and 4 minutes (Frimer & Skitka, 2018a). To estimate the sample necessary to produce effect size estimates with a narrow enough confidence interval to distinguish between the two closest predictions (4 minutes), we drew (random) samples of various sizes from the data from Study 1 and plotted the width of the 95% confidence intervals against the sample size in Figure S3. The result was a power curve with the best-fit formula *CI* = 3383.9 × *N*^-0.525^. Therefore, the needed sample size is *N* = (4/3383.9)^1/-0.525^ ~377,000. Even aiming to distinguish Chen and Rohla’s estimate of 30-50 minutes from the null would require ~8,000 participants. These samples are not feasible using crowdsourcing.

**Figure S3**. Power curve from Study 1. Each dot represents the width of the 95% CI from a random subsample.

**Table S1**. Results from regression analyses testing whether diversity predicts Thanksgiving dinner duration with outliers (> 3 *SD*s from the *M*) of dinner duration removed. In Study 1, we removed 9 outliers (1.5%), which reduced the kurtosis from 3.77 to 0.53. In Study 2, we removed 29 outliers (2.6%), which reduced the kurtosis from 3.30 to 2.04.

| **Study 1: Attitudes Toward Trump** | | |  |  |
| --- | --- | --- | --- | --- |
|  | **Predictor** | **B [95%CI]** | **β** | ***p*** |
|  | (Constant) | 298 [280, 316] |  | <.001 |
|  | Diversity | 22 [-32, 76] | .034 | .421 |
| **Study 2: Attitudes Toward Trump** | | |  |  |
|  | **Predictor** | **B [95%CI]** | **β** | ***p*** |
|  | (Constant) | 355 [336, 374] |  | <.001 |
|  | Diversity | 15 [-42, 73] | .016 | .599 |
| **Study 2: Attitudes Toward Impeachment** | | | |  |
|  | **Predictor** | **B [95%CI]** | **β** | ***p*** |
|  | (Constant) | 370 [349, 391] |  | <.001 |
|  | Diversity | -28 [-83, 27] | -.031 | .319 |
| **Study 2: Party Identification** | | |  |  |
|  | **Predictor** | **B [95%CI]** | **β** | ***p*** |
|  | (Constant) | 348 [323, 373] |  | <.001 |
|  | Diversity | 38 [-35, 112] | .031 | .308 |

**Table S2**. Results from regression analyses testing whether diversity is related to Thanksgiving dinner duration in a non-linear manner.

| **Study 1: Attitudes Toward Trump** | | | | |
| --- | --- | --- | --- | --- |
|  | **Predictor** | **B [95%CI]** | **β** | ***p*** |
|  | (Constant) | 313 [291, 335] |  | <.001 |
|  | Diversity | -37 [-193, 118] | -.050 | .637 |
|  | Diversity^2^ | 85 [-158, 327] | .073 | .494 |
| **Study 2: Attitudes Toward Trump** | | | | |
|  | **Predictor** | **B [95%CI]** | **β** | ***p*** |
|  | (Constant) | 385 [361, 409] |  | <.001 |
|  | Diversity | 80 [-131, 291] | .069 | .457 |
|  | Diversity^2^ | -201 [-564, 161] | -.101 | .277 |
| **Study 2: Attitudes Toward Impeachment** | | | | |
|  | **Predictor** | **B [95%CI]** | **β** | ***p*** |
|  | (Constant) | 401 [373, 429] |  | <.001 |
|  | Diversity | -50 [-208, 108] | -.046 | .533 |
|  | Diversity^2^ | -10 [-215, 195] | -.007 | .924 |
| **Study 2: Party Identification** | | | | |
|  | **Predictor** | **B [95%CI]** | **β** | ***p*** |
|  | (Constant) | 382 [344, 421] |  | <.001 |
|  | Diversity | 91 [-156, 338] | .060 | .468 |
|  | Diversity^2^ | -231 [-604, 143] | -.101 | .226 |

Study 2

**Figure S4**. Histogram of Thanksgiving 2019 dinner arrival and departure times.

**Figure S5**. Histogram of Thanksgiving 2019 dinner durations.

**Table S3**. Frequency counts of how far participants traveled to reach Thanksgiving Dinner.

| **Distance (miles)** | **Frequency** |
| --- | --- |
| 0 | 46.5% |
| 1 | 3.4% |
| 2 | 3.8% |
| 5 | 10.7% |
| 10 | 10.9% |
| 20 | 12.6% |
| 50 | 5.9% |
| 100 | 2.8% |
| 200 | 2.2% |
| 500 | 0.6% |
| 1000 | 0.3% |
| 2000 | 0.1% |

**Table S4**. Frequency counts of how long it took participants to travel to reach Thanksgiving Dinner.

| **Time (minutes)** | **Frequency** |
| --- | --- |
| <30 | 65% |
| 60 | 10% |
| 90 | 9% |
| 120 | 2% |
| 150 | 3% |
| 180 | 1% |
| 210 | 2% |
| 240 | 1% |
| 270 | 2% |
| 300 | 1% |
| 330 | 1% |
| 360 | 1% |

**Figure S6**. Mediation model assessing whether an unpleasant and/or engaging social atmosphere helped explain why political diversity, defined with respect to political party affiliation, might not be associated with shorter Thanksgiving dinners. Dinner duration was operationalized as the residuals while controlling for contextual factors (see Table 6). † *p* < .10, ** p < .01.


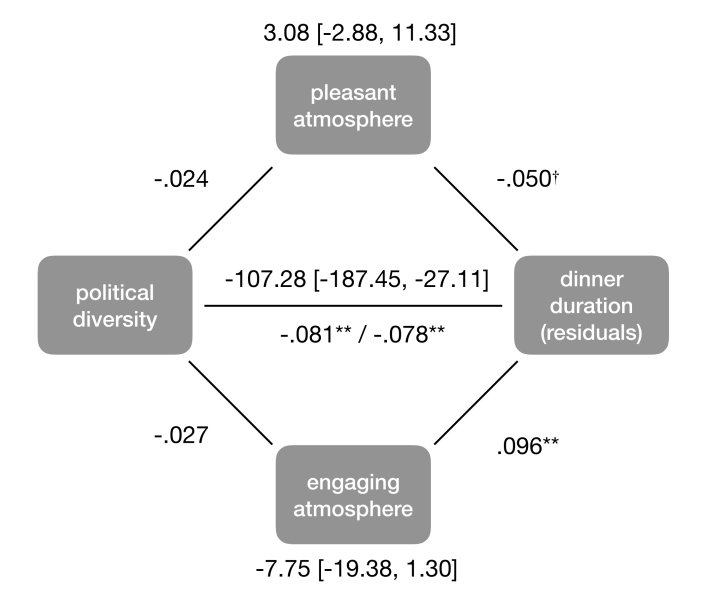


**Mega-Analysis**

In the main text, we used our pre-registered operationalized political diversity, which averaged the political difference between each and every dyad at the dinner. That is, the measure of diversity included not only differences between the participant in our study and each of the other guests, but also among each of the other guests. Chen and Rohla (2018) operationalized diversity in terms of political differences between the subject and the host. To more closely mirror Chen and Rohla’s (2018) operationalization, we computed a political diversity measure as the difference between the participant’s attitudes and the average attitudes of the rest of the dinner. Using this definition, we found that diverse dinners were 2 minutes longer, 95%CI = [-16, 19], β = .003, *p* = .859, in a zero-order analyses and 10 minutes shorter 95%CI = [26 minutes shorter,6 minutes longer], β = -.019, *p* = .217, in a conditioned analyses. Neither of these effects replicate Chen and Rohla (2018) using either criterion.
